# The Effect of Colored and White Light on Growth and Phycobiliproteins, Chlorophyll and Carotenoids Content of the Marine Cyanobacteria *Phormidium* sp. and *Cyanothece* sp. in Batch Cultures

**DOI:** 10.3390/life12060837

**Published:** 2022-06-04

**Authors:** George N. Hotos, Theodoros I. Antoniadis

**Affiliations:** Plankton Culture Laboratory, Department of Animal Production, Fisheries and Aquaculture, University of Patras, 30200 Messolonghi, Greece; antoniadistheodoros@hotmail.com

**Keywords:** cyanobacteria, *Phormidium*, *Cyanothece*, culture growth, light, chlorophyll, carotenoids, phycobiliproteins

## Abstract

Two local marine cyanobacteria, *Phormidium* sp. and *Cyanothece* sp., were batch-cultured under 18–19.5 °C, at 40 ppt salinity, using white LED light of low (40 μmol photons/m^2^/s) and high (160 μmol/m^2^/s) intensity and, additionally, blue, green and red LED light. Yield was highest in high white light in both species (2.15 g dw/L in *Phormidium,* 1.47 g/L in *Cyanothece*), followed by green light (1.25 g/L) in *Cyanothece* and low white and green (1.26–1.33 g/L) in *Phormidium*. Green light maximized phycocyanin in *Phormidium* (0.45 mg/mL), while phycoerythrin was enhanced (0.17 mg/mL) by blue light and allophycocyanin by all colors (~0.80 mg/mL). All colors maximized phycocyanin in *Cyanothece* (~0.32 mg/mL), while phycoerythrin and allophycocyanin peaked under green light (~0.138 and 0.38 mg/mL, respectively). In *Phormidium*, maximization of chlorophyll-a (9.3 μg/mL) was induced by green light, while total carotenoids and b-carotene (3.05 and 0.89 μg/mL, respectively) by high white light. In *Cyanothece*, both white light intensities along with green maximized chlorophyll-a (~9 μg/mL) while high white light and green maximized total carotenoids (2.6–3.0 μg/mL). This study strongly indicates that these cyanobacteria can be cultured at the first stage under white light to accumulate sufficient biomass and, subsequently, under colored light for enhancing phycobiliproteins.

## 1. Introduction

Cyanobacteria comprise a vast assemblage of prokaryotic microalgae that are found in ecosystems highly diversified in terms of extreme conditions prevailing both seasonally and nutritionally [1]. They colonize every habitat due to their ability to adapt and cope with a great variation of light intensity and spectra, temperature, and nutrient availability [2,3,4]. They possess oxygenic photosynthesis along with eukaryotic algae and share with them the same basic and accessory pigments (chlorophyll-a and carotenoids, respectively) [5]. Additionally, cyanobacteria are equipped with unique photosynthesis, aiding pigments of a proteinaceous structure named phycobiliproteins (phycocyanin, allophycocyanin and phycoerythrin, blue-, bluish-green- and red-colored, respectively), which impart to them their characteristic bluish-greenish tint, hence their common name “blue-green” algae [6,7,8]. All the above-mentioned pigments that function in the harnessing of light energy for algae to transform CO_2_ to sugars in order to increase their biomass have tremendous significance for certain sectors of bioindustry, as healthy ingredients for humans and animals, and for fine chemicals [9,10]. In particular, the biliprotein phycocyanin, a water-soluble pigment with wide and continuously expanded uses in biology, medicine and the food industry [6,11], presents front-line exploitable potential. Based on rough statistics [12,13], its world market value is estimated to be USD 50 million per annum with a commercial value from USD 0.13 to 25 per mg, depending on its purity level from food grade (the lowest) to analytical grade (the highest) [14].

Although various cyanobacterial species can widely be mass cultured in farms for various purposes [15], the lion’s share is held by the genus *Arthrospira* (*Spirulina*) [13,16], overshadowing initiatives for other similar exploitable species. As cyanobacteria offer unique advantages for culture such as independence from nitrogenous nutrient sources due to the atmospheric nitrogen fixation for some of them [15], their efficient light capture machinery [17] or the self-aggregation of their cell mass (e.g., *Phormidium*), a property much appreciated in culture procedure [18,19], research on their mass production is worth trials.

**Figure 1:**
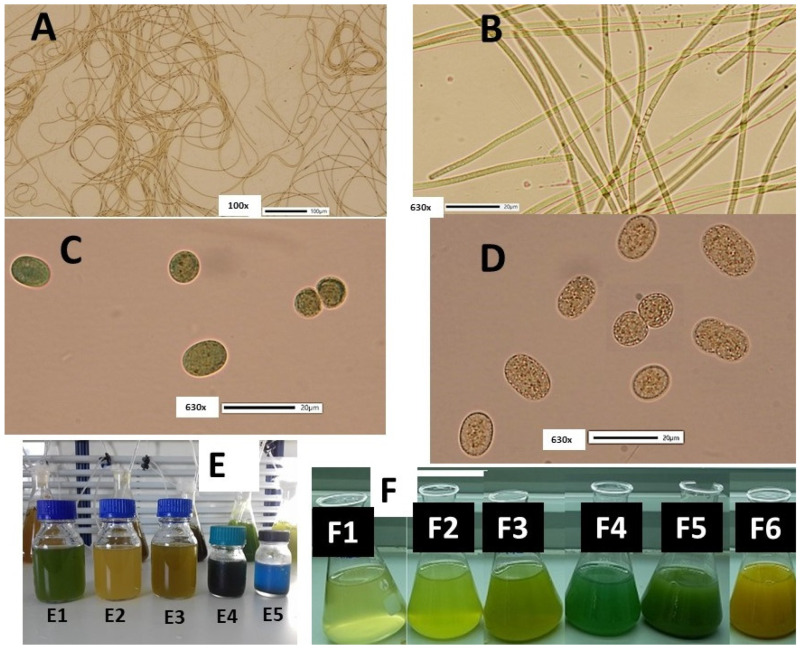
(**A**) *Phormidium* sp. dense mass of filaments at 100x magnification. (**B**) Filaments at 630× magnification. (**C**) Cells of *Cyanothece* sp. colored greenish on the 8th day of culture, 630×. (**D**) Cells of *Cyanothece* sp. colored yellowish on the 18th day of culture, 630×. (**E**) Characteristic colors of samples of *Phormidium* from different stages of culture and treatment; (**E1**) from culture in exponential phase (8th day); (**E2**) from stationary phase (13–15th day); (**E3**) from old culture (>18th day); (**E4**) a dense after centrifugation sample from the exponential phase to be frozen; (**E5**) the same sample after thawing exhibited profound phycocyanin release. (**F**) *Cyanothece* sp. Color of its cultures exhibiting intense differentiation depending on the age of culture, (**F1**) 2 days, (**F2**) 5 days, (**F3**) 11 days, (**F4**) 13 days, (**F5**) 15 days and (**F6**) 18 days.

For all microalgae (prokaryotic and eukaryotic), the culture productivity, on the one hand, and their biochemical composition (photosynthetic pigments included), on the other, are greatly affected by physicochemical and nutritional factors prevailing in their cultivation [20]. Among them, light is of paramount importance, as both the quantity (intensity) and quality (color) of the visible spectrum utilized for photosynthesis affect profoundly the productivity of the culture and the biochemical composition of the cells [21,22,23]. As the increased interest of the world market for carotenoids from natural sources [24] and biliproteins [6,13,25] substantiate the experimentation on optimizing their production, the parameter of light emerges as a key-role factor for accomplishing that [14,19]. Although cyanobacteria were limitedly mentioned in the recent review work of [23], where the influence of light on microalgae was reviewed, there are several studies that focused on the effect of light intensity and its color on growth and cell constituents of several cyanobacterial species, mainly *Arthrospira* [14,26,27,28,29,30,31], *Dolichospermum* (*Anabaena*) [32], *Nostoc* [32], *Phormidium* [18,19] and various other species [33,34,35,36,37]. On this ground, we examined the effect of light on two locally isolated (saltworks of Messolonghi, W. Greece, [38]) cyanobacteria, the coccoid N-fixing *Cyanothece* sp. and the filamentous non N-fixing *Phormidium* sp. (Figure 1). As both of them exhibited potential for fast growth in a wide range of salinity and light intensity in preliminary or elaborated cultures and, additionally, this *Phormidium* strain proved to be a profound phycocyanin producer [19], we decided to deepen our investigation into their response to different light regimes. Both of them exhibit intense chromatic adaptation as a result of the ageing of culture in combination with light intensity, a phenomenon manifested in cyanobacteria [39,40], which triggered our interest in monitoring their pigment content in batch culture. Besides *Spirulina* (cyanobacterium) and *Porphyridium* (red algae) biliprotein commercial production, which occupies the bulk of the business [13], there is always space for exploring other cyanobacterial species for the same purpose and additionally for chlorophyll and carotenoids. As, to the best of our knowledge, there are no studies concerning the effect of colored light on species of *Cyanothece* and *Phormidium*, our objective in the present study was to fill this gap by examining the influence of white and color light on their growth and pigment content.

## 2. Materials and Methods

**Figure 2:**
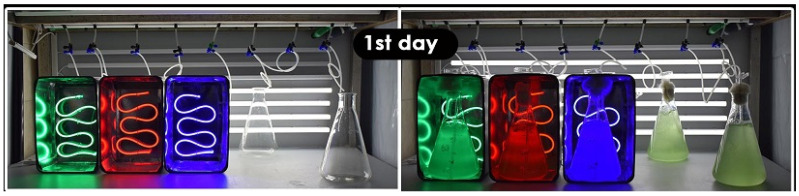
A set of the colors’ configuration setup used in the experimentation before (**left**) and after (**right**) the start of the culture.

The strains of *Phormidium* sp. and *Cyanothece* sp. used came from the hypersaline ponds of the nearby saltworks of Messolonghi. After serial dilutions and repeated renovations of cultures in 40 ppt salinity, eventually, they were isolated as monospecific cultures to be used thereafter. The experiment took place at 18–19.5 °C in an 18.000 BTU air-conditioned laboratory. Two-liter autoclaved glass conical Erlenmeyer flasks were used in triplicate for each light filled up to the 1.85 L mark with sterilized sea water of 40 ppt salinity enriched with nutrients according to Walne’s formula (given at the end of this section). At the start of the batch culture, the inoculum of each cyanobacterium was 150 mL (to 1850 mL of 40 ppt water), taken from the mother cultures, which were maintained in exponential phase at 40ppt salinity. In every case of salinity adjustments, this was performed with sterilized distilled water. Two light types were used: white light emitted by an array of 20 Watt 1600 Lm LED lamps and colored light by blue, green and red LEDs (BarisLight neon silicon strip 9 W/m, 400 Lm/m), each housed in a properly constructed box, the interior of which did not receive other light than the assigned color (Figure 2). In the case of white light, the desired intensities of 40 and 160 μmol/m^2^/s were attained by properly placing the vessels at the proper distance from the lamps. Intensity was measured at the middle of the outer surface of the vessels by means of a luminometer (BIOBLOCK LX-101). In the case of colored lights, the light intensities measured at the surface of the vessels positioned 1cm away from the LED stripes were 54, 56 and 60 μmol/m^2^/s for red, green and blue LEDs, respectively. Illumination in all cases was continuous (24 h L:0 hD). The cultures were kept in suspension by means of bubbling air (with its natural CO_2_ level) through 2 mL glass pipettes (one in every vessel) at a rate of half culture volume/min. The pipettes were connected through sterilized plastic hoses to the 0.45 μm filtered central air supply system fed by a blower.

Culture progress was monitored spectrophotometrically by measuring the absorbance of a small sample at 750 nm using a Shimadzu Uvmini-1240 UV-visible spectrophotometer. The measurements were transformed to dry biomass per volume (g/L) using a proper equation derived from calibration curves of dry weight vs. absorbance (Supplementary Material, S1) using previous dense cultures of each species with serial dilutions and, additionally, more couples of values from culture samples taken every 3 days. Based on the daily records of absorbance and the constructed growth curves of the cultures (Supplementary Material, S1), the days for the dry weight estimation were defined.

Dry weight was calculated by filtering a known amount of culture through 0.45 μm GF/C filters in a vacuum pump (Heto-SUE-3Q). The filters were next washed with ammonium formate for removing salts and then placed in an oven at 100 °C for 2 h. After that, they were weighted to the 4th decimal, and the dry weight was calculated as g/L. In every daily sample taken from the culture, the pH was also measured using a digital pH meter (HACH-HQ30d-flexi). *Cyanothece* culture lasted 18 days, and *Phormidium* culture lasted 21, days due to the signs of senescence they exhibited on these days. There was no point to continue furthermore as what they had to exhibit they exhibited already (in the most fulfilling way concerning pigment content) during the previous days. The maximum specific growth rate (SGR) was estimated during the exponential phase of the culture’s growth curve (from the 3rd till the 16th day in *Phormidium*, from the 3rd till the 15th day in *Cyanothece*) using the equation:

SGR = (lnC_2_ − lnC_1_)/(t_2_ − t_1_) (1)

where C_1_ and C_2_ stand for g D.W. of cells on days t_1_ and t_2_, respectively (t_2_ > t_1_).

From the above equation, the generation time T_g_ of the culture was calculated as days for doublication using the formula:

T_g_ = 0.6931/SGR (2)

Chlorophyll-a and total carotenoids were extracted from centrifuged culture samples with absolute methanol, and their concentration (μg/mL) was calculated spectrophotometrically using the equations [41]:

chl-a = 12.9447 (A665 − A720)(3)

total carot. = [1000 (A470 − A720) − 2.86 chl-a]/221(4)

Beta-carotene (b-carotene) was extracted using a 2:4 mixture of hexane/methanol using a slightly modified recipe of [42], and its concentration (μg/mL) was calculated spectrophotometrically using the equation:

(5) $$b-\mathrm{carot}.=\left(\frac{A453-A665\;}{3.91}\right)\times 3.657\times 2\times {\;D}_{\;}$$

with (A453 − A665/3.91): absorbance of b-carotene corrected for chlorophyll contamination, 3.657: calibration factor derived from HPLC analysis of b-carotene concentration, 2: amount of mL hexane used, D: the dilution factor for hexane used for spectrophotometric measurement in cases when the initial absorbance exceeds the value of 2.

Phycocyanin (PC), allophycocyanin (APC) and phycoerythrin (PE) content were extracted by freezing (−20 °C) for 24 h a concentrated known amount of culture in 0.1 M sodium phosphate buffer (pH 7.1) as the solvent at a ratio of 1:10 (algal mass: solvent) and then thawing at 4 °C in darkness. The freezing/thawing procedure was repeated for two consecutive days. The sample’s slurry was then centrifuged at 3000 rpm for 5 min, and the supernatant was measured spectrophotometrically to calculate the amount of the pigments (in mg/mL) using the equations [43]:

(6) $$\mathrm{PC}=\frac{A_{615}-0.474{\;A}_{652}\;}{5.34}$$

(7) $$\mathrm{PE}=\frac{A_{562}-[\left(2.41\mathrm{PC}\right)-\left(0.849\mathrm{APC}\right)\;}{9.62}$$

(8) $$\mathrm{APC}=\frac{A_{652}-0.208{\;A}_{615}\;}{5.09}$$

From the above equations, the yield of phycocyanin in mg PC/g dry weight was calculated using the equation [44]:

(9) $$\mathrm{PCyield}=\frac{\mathrm{PC}\;\left(\frac{\mathrm{mg}}{\mathrm{mL}}\right)\times \;V\;\left(\mathrm{mL}\right)\;}{D.W.\;\left(g\right)}$$

where:

PCyield = mg of phycocyanin per g algal dry weight;

V = volume of solvent used (mL);

D.W. = grams of dry weight of the algal mass used;

The purity of phycocyanin was recorded as the ratio of absorbance of 620 to 280 nm [45].

Statistical treatment of the different variables was performed with ANOVA and Tukey’s test for comparison of the means at the 0.05 level of significance using the free PAST3 software.

Walne’s medium formula is comprised of 3 final stock solutions A, B and C with the appropriate dilution of substances in 1 L of distilled water, from which 1 mL of each is used per liter of culture water. Solution A is metals, NaNO3 300 g, NH4Cl 20 g, KH_2_PO_4_ 30 g. Solution B is trace elements ZnSO_4_.H_2_O 30 g, CuSO_4_.5H_2_O 25 g, CoSO_4_.7H_2_O 30 g, MnSO_4_.H_2_O 20 g. Solution C is vitamins, B_12_ 100mg, biotin 100 mg, thiamine 10 mg.

## 3. Results

### 3.1. Biomass

**Figure 3:**
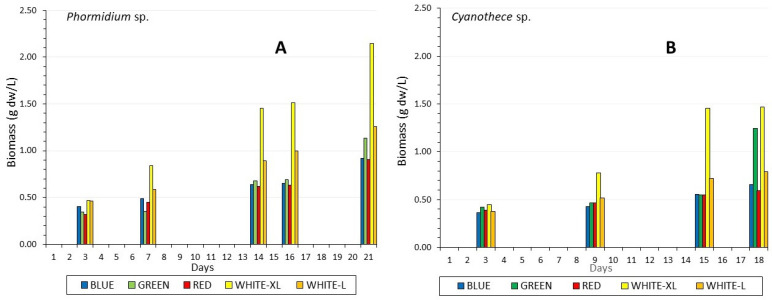
Biomass production as g dry weight/L through the culture period in: (**A**) *Phormidium* sp. and (**B**) *Cyanothece* sp. WHITE-XL = white light of 160 μmol/m^2^/s, WHITE-L = white light of 40 μmol/m^2^/s, BLUE, GREEN, RED = specific color light used.

In every light regime, the biomass increased during the culture period in both species (Figure 3), and the increase was much higher in white light, in which the higher intensity (160 μmol/m^2^/s) values were almost double those of low intensity (40 μmol/m^2^/s) after the 7th and 9th day in *Phormidium* and *Cyanothece*, respectively. *Phormidium* grew faster than *Cyanothece* as evidenced also by the higher specific growth rate and the corresponding lower generation time (Table 1), reaching a maximum of 2.15 g dw/L on the 21st day, higher than the 1.47 g dw/L of *Cyanothece* on the 18th day. In both species, the growth was almost identical in all colors, but from the 15–16th day, green color induced abruptly higher growth than blue and red, reaching 1.25 and 1.14 g dw/L on the 18th and 21st day in *Cyanothece* and *Phormidium*, respectively, values statistically different (*p* < 0.005) from the respective ones of all other colors. However, the specific growth rate fluctuated to low values for *Phormidium* (max. 0.131 in green light in *Phormidium*) and even lower in *Cyanothece* (max. 0.098 in high white light). pH fluctuated throughout the culture period between 8.3 and 9.5 with remarkably higher values of greater than 9.0 in high white light in both species.

### 3.2. Chlorophyll and Carotenoids

**Figure 4:**
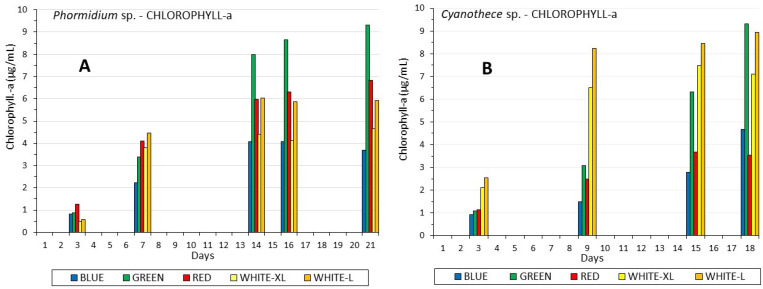
The development of chlorophyll-a content in μg/mL along the culture period of (**A**) *Phormidium* sp. and (**B**) *Cyanothece* sp.

Significant differences were found in chlorophyll-a content between Phormidium and Cyanothece (Figure 4). In both species, the maximum chlorophyll-a content was ~9 μg/mL, but recorded at different days of the culture (earlier in Cyanothece) and with greater and statistically significant differences among the colors. In both species, higher chlorophyll content was induced by white low light (40 μmol/m^2^/s) compared to white high light (160 μmol/m^2^/s, *p* < 0.005). However, the highest values in both species were recorded in green light, and this phenomenon was much more pronounced in Phormidium after the 14th day with values statistically greater (*p* < 0.005) than any other light, while in Cyanothece, the green value peaked later on the 18th day and was statistically equal to that of white low light (*p* > 0.005). Worth mentioning is also the fact that, in both species, after about 7 days, the ratio of the values of chlorophyll-a between low and high white light remained almost the same.

**Figure 5:**
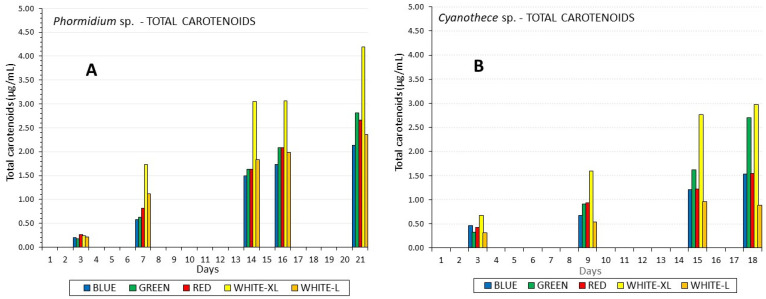
The development of total carotenoids content in μg/mL along the culture period of (**A**) *Phormidium* sp. and (**B**) *Cyanothece* sp.

In contrast to chlorophyll, a much higher total carotenoids content was induced by high white light compared to low light in both species, 4.2 μg/mL in *Phormidium* on the 21st day and 3.0 μg/mL in *Cyanothece* on the 18th day (Figure 5). All other colors had a quite uniform influence on carotenoids content, exhibiting a steady, slow increase in the course of the culture period, but with red and green in *Phormidium* and green in *Cyanothece* on the 21st and 18th day, respectively, their influence peaking at levels significantly higher (*p* < 0.005) than their blue and red counterparts, respectively. The ratio of chlorophyll to total carotenoids was maximum in all lights during the early phase of the culture (Table 2), peaking on the 7th day in *Phormidium* with the highest value of 5.44 for the green light and the lowest 2.2 for the high white light. In *Cyanothece*, the chl.:carot. ratio presented quite similar values among colors throughout the culture period with the lower values in blue light (2.0–3.0) and the highest in low white light (8.0–15.0). b-carotene in *Phormidium* presented its highest value (0.946 μg/mL) in white high light, followed by green light (0.783 μg/mL), red and white low light ((0.64 μg/mL) and blue light (0.49 μg/mL). The total carotenoids: b-carotene ratio exhibited rather small differences among treatments ranging from 3.58 to 4.44, with higher values recorded on the final days of the culture in all light regimes. Unfortunately, in *Cyanothece*, due to a technical problem, there were no data for b-carotene.

### 3.3. Phycobiliproteins

**Figure 6:**
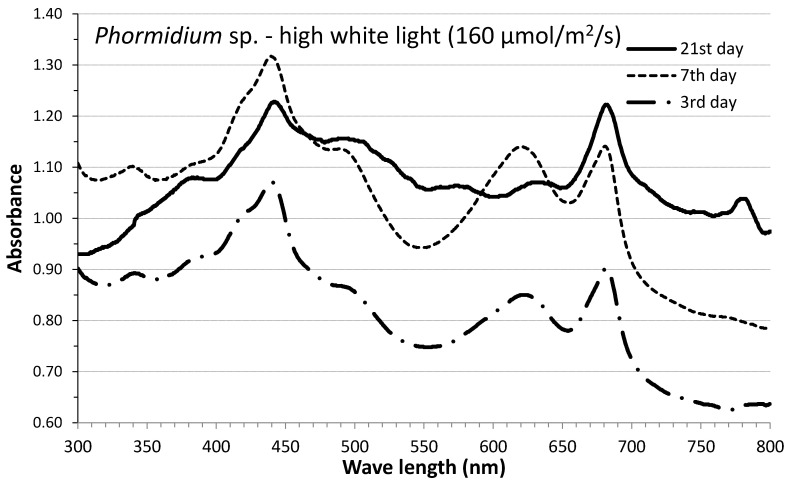
Absorption spectra of culture samples of *Phormidium* sp. under high-intensity white light recorded at differed culture days.

**Figure 7:**
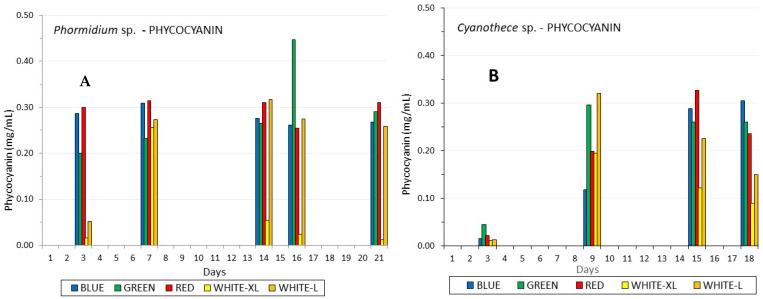
The development of phycocyanin content in mg/mL along the culture period of (**A**) *Phormidium* sp. and (**B**) *Cyanothece* sp.

**Figure 8:**
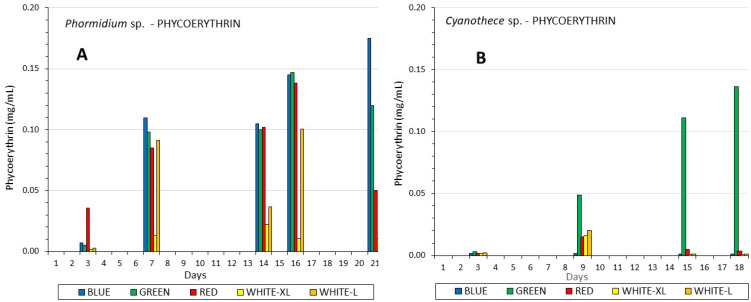
The development of phycoerythrin content in mg/mL along the culture period of (**A**) *Phormidium* sp. and (**B**) *Cyanothece* sp.

Great variation in every particular phycobiliprotein content was recorded among the lights used and among the days of the culture period. As illustrated in the exemplified absorption spectra diagrams of high white light for the start, middle and final stages of the culture of Phormidium (Figure 6), the relative content of chlorophyll-a (440 and 685 nm), carotenoids (500 nm), phycoerythrin (580 nm) and phycocyanin (625 nm), as evidenced by their characteristic peaks, was greatly modified along with culture age. While the peaks of chlorophyll-a remained stable, phycocyanin was maximized on the 7th day and phycoerythrin on the 21st day in accordance with the data presented in Figure 7 and Figure 8 with respect to the phycocyanin and phycoerythrin content of high white light in Phormidium.

Phycocyanin content was much influenced by all colors in Phormidium (Figure 7A), and from the first days of the culture, its concentration reached values close to 0.3 mg/mL, which peaked on the 16th day (0.46 mg/mL) under green light, statistically different (*p* < 0.005) from all other lights. Thereafter, on the 21st day, the influence of the green light diminished. Red and blue light, except for the 16th day, also induced, comparable to red and low white light, the production of phycocyanin. Apart from colored light, the low white light after the seventh day induced much greater production of phycocyanin compared to high white light.

In Cyanothece (Figure 7B), the increase of phycocyanin was much more delayed than in Phormidium from the very low values for all lights of ~0.03 mg/mL on the 3rd day to values around 0.3 mg/mL on the 7th day by low white and green lights. Thereafter, the influence of white light diminished and red and blue colors reached the values of green in the region of 0.3 mg/mL. As a rough approximation, we can claim that in both species, phycocyanin production much favored the colored light (especially in Cyanothece), reaching ~0.3 mg/mL.

Phycoerythrin exhibited a totally different image than phycocyanin between the two species, although in a generalized consideration, the maximum content was in the region of 0.15 mg/mL for both species, but with a totally different pattern for each of them. In Phormidium (Figure 8A)m after the seventh day, all colors induced much more content (*p* < 0.005) than every kind of white light, and finally, on the 21st day, blue light (*p* < 0.005, induced 0.175 mg/mL, a value considerably higher than any other light. In Cyanothece (Figure 8B), an astonishing and overwhelming influence on phycoerythrin content was recorded using green light, which greatly surpassed all kinds of light (*p* < 0.005) throughout the culture period, reaching 0.13 mg/mL on the 18th day. In Cyanothece, in contrast to the above-mentioned case of phycocyanin for both species and phycoerythrin in Phormidium, high and low white lights had a negligible influence on the induction of phycoerythrin production.

**Figure 9:**
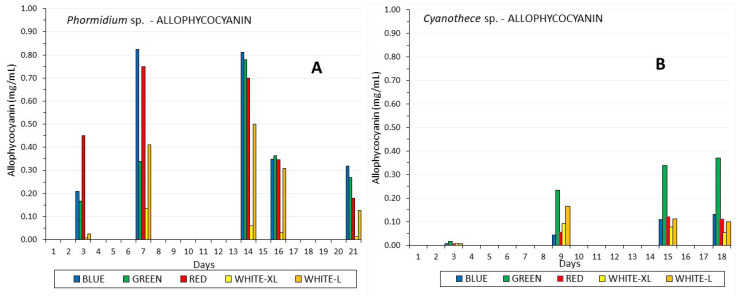
The development of allophycocyanin content in mg/mL along the culture period of (**A**) *Phormidium* sp. and (**B**) *Cyanothece* sp.

A pattern remarkably similar to phycoerythrin of the difference between the two species was also recorded in the case of allophycocyanin (Figure 9), as firstly, its content was much greater in Phormidium (max. 0.83 mg/mL) compared to Cyanothece (max. 0.37 mg/mL) and, secondly, its maximization in Cyanothece was also induced (similar to phycoerythrin) by green light far more than any other kind of light (*p* < 0.005). The two species differed in that allophycocyanin production in Phormidium was favored by all colors over white light instead of only by green, as in Cyanothece.

Considering the total content of all biliproteins (Table 2), no clear pattern could be detected, neither among days nor among light treatments. The higher value in Phormidium was recorded in green light on the 14th day (1.145 mg/mL with 0.374 mg/mL on the 3rd day) and the lowest in high white light on the 3rd day (0.026 mg/mL and maximum 0.136 mg/mL on the 14th day). In contrast to Phormidium, Cyanothece exhibited a clear pattern of a continuous increase of total biliproteins in all light treatments as the culture proceeded. Green light exhibited over all other treatments a maximal value of 0.768 mg/mL on the 18th day (with minimal 0.065 mg/mL on the 3rd day) and, for both white lights, the lowest values in comparison to all colors (~0.02 min. to 0.250 mg/mL max.).

Totally different ratio patterns were recorded among the two species (Table 2) concerning the ratio of phycocyanin (PC) to phycoerythrin (PE). In Phormidium, the PC:PE ratio exhibited the highest values on the third day with maxima in the green and blue light (39.3 and 40.3, respectively) and minima in the high white and red light (8.4), and the ratios continuously decreased to end up on the 21st day with values much smaller, 1.1 for high white light, 1.5 for blue, 2.4 for green, 6.2 for red and 10.3 for low white light, which, with an initial value of 18.1, along with red light (from 8.4 to 6.2), exhibited a lower decrease. In Cyanothece, contrary to Phormidium, the PC:PE ratio exhibited a reverse trend with low values in the beginning (third day), 6.3–6.6 for both white lights, 9.4 for blue and ~15.0 for red and green. With the advance of the culture in all lights, the ratios increased gradually to reach much higher values on the 18th day from 67.7 for red to 125–150 for white lights and 305 for blue light. A remarkable exception was the green light, which, contrary to all other lights, exhibited a constant decrease in its values from 15.2 on the 3rd day to 1.9 on the 18th day.

The ratio of phycocyanin to allophycocyanin (PC:APC) fluctuated with very low, but rather similar values (Table 2) throughout the culture period in Cyanothece in all lights (0.7–3.5) and was even lower in Phormidium (0.3–2.0).

The ratio of total phycobiliproteins (PBP) to chlorophyll and total carotenoids (PBP:Chl and PBP:Tcar, respectively) exhibited quite a similar pattern between the two species (Table 2). Both PBP:Chl and PBP:Tcar steadily increased from the 3rd day till the 9th and then decreased. In Phormidium, the PBP:Chl values in general were much higher in all lights than Cyanothece (e.g., 616 vs. 109 for blue light), and this was further exaggerated in PBP:Tcar (e.g., 1735 vs. 331 also for blue light).

In terms of yield of phycocyanin and phycoerythrin, the two species exhibited quite uniform values (Table 2), ranging from 18.52 to 31.76 mg/g dw phycocyanin in blue and green light, respectively, in Phormidium and from 18.34 to 34 mg/g dw for high white and red light, respectively, in Cyanothece. Considerably lower values were recorded for phycoerythrin in both species, but overall, Phormidium produced more. In both species, the maximal amount was produced under green light (10.44 and 12.07 mg/g dw for Phormidium and Cyanothece, respectively), and while in Cyanothece, the value for green far exceeded any of the other colors, in Phormidium, blue and red induced similar yields to that of green (10.62 and 10.87 mg/g dw, respectively).

**Figure 10:**
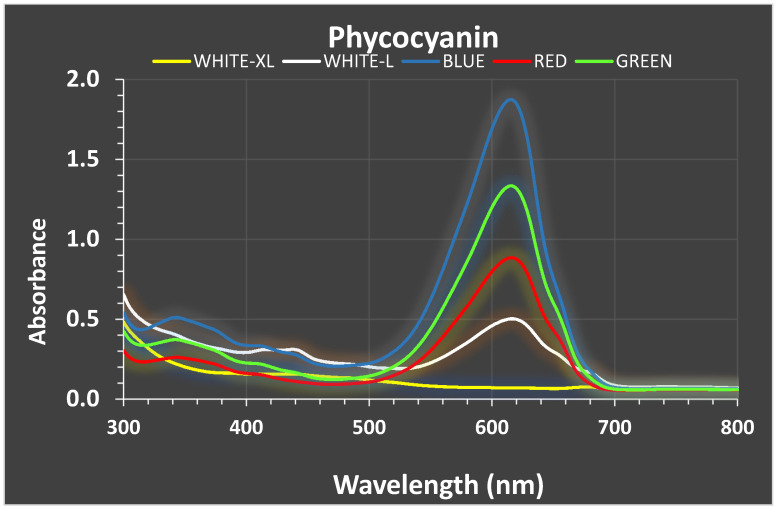
An array of absorption spectra of phycocyanin crude extracts on the 14th day of culture of *Phormidium* sp. under the different light treatments.

The purity of phycocyanin tested by recording the ratio of absorbance of crude extract between the wave lengths of 620 and 280 nm, considering the value of 0.7 as the border of satisfactory purity (above) and poor (below), was in most cases above 0.7 in both species. An example is given in Figure 10, where the absorption spectra of the crude extract of phycocyanin from samples of the 14th day from all colors in Phormidium were put together. It is evident that the curves present a greater height between the peaks of phycocyanin at 625 nm and the region below 400 nm (characteristic of other proteins) in all colors, as compared to low white light and the absence of purity of high white light. In general, blue light proved the most efficient with values over 1.5, low white light just on the edge (~0.7) and high white light very low (~0.2).

## 4. Discussion

As cyanobacterial cultures are greatly affected in terms of the output of biomass and pigments (let alone other constituents) by the conditions prevailing during the culture period and light is of paramount importance [46,47,48], the proper adjustment of light quantity and quality is perhaps the most crucial task to be accomplished. As indicated in Hotos and Avramidou, 2021 [49], a sound approach for optimizing the production of value-added algal products is to establish first realistically attainable conditions of energy cost and temperature range. Only if the species under investigation can grow well in the not-so-high temperature range of ~20–23 °C instead of the extreme 30–36 °C frequently encountered in culture optimization studies (e.g., [35,50,51]), there are real perspectives for the economics of a culture aiming to maximize pigments’ output by testing light manipulation. In this respect, the present work follows that of Hotos, 2021 [19], in which *Phormidium* sp., a filamentous non diazotrophic species was found as a very promising species for mass production in moderate temperature regime (~22 °C). Now, *Phormidium* and *Cyanothece* sp., a coccoid single-celled diazotrophic species [52], was tested under different light regimes as both preliminary cultures exhibited high growth rates. Both of them exhibited at late stages of culture intense chromatic adaptation, a feature much pronounced in cyanobacteria [53]. Their response to different colors of light in combination with the alterations of their biliprotein content during growth, which finally is manifested in the turning of their green coloration in the exponential phase to yellow-green in the stationary phase with various hues of green in between, leaves no doubt that both belong to Group II cyanobacteria according to Tandeau de Marsac, 1977 [54]. Their high content of phycoerythrin at late stages (especially in *Cyanothece*), which is a very flexible biliprotein in adaptation to changing environmental conditions (especially light) [55], imparts to them their change of coloration and broadens the range of light-harvesting capability in the green area of the light spectrum [56,57].

Reviewing the literature on the pigment content in cyanobacteria, the situation is perplexed and puzzling as there are highly variable and, sometimes, contradicting data. However, two things in general can be distilled from the overall bulk of the findings. First, the growth of cyanobacteria under different regimes of light quality is highly variable among species (e.g., [58,59,60]), and second, as there is not a fixed ratio of pigments in the cellular volume among the species, an open wide field of experimentation for optimizing yields exists. The first prerequisite of optimization is the satisfactory production of enough biomass. Without producing enough algal mass, the quest for maximizing the production of pigments is meaningless. In this respect, the role of light is catalytic, and we need to compromise between two opposite directions of adjusting light intensity. This is because cyanobacteria grow faster in continuous high light intensities [8,19], and the findings of the present study support strongly this for both *Phormidium* with 1.52 g dw/L in high light of 160 μmol/m^2^/s as compared to 1 g/L in low light of 40 μmol/m^2^/s and *Cyanothece* with 1.62 and 1.02 g/L, respectively. On the other hand, cyanobacteria can cope effectively with low light intensities due to their low energy requirement for maintenance and their unique flexible pigment composition, which, by broadening the spectrum of available low light, can balance the photon energy between PS I and PS II [51,61]. As biliproteins constitute 20–40% of total proteins in cyanobacteria [57,62] and outnumber greatly chlorophyll and carotenoids, their profound increase at low light intensities for extracting the maximum of photons available comes as no surprise [50,63,64]. This was recorded in all treatments of low white light for both species in the present study and for all light colors used and proves that the underlying mechanism is not only the wavelength of the light that triggers a particular cellular response, but the intensity per se, as the intensities of the colored light used were less than 60 μmol/m^2^/s. Furthermore, we recorded an increase in the amount of biliproteins as the culture growth period lengthened. Due to their high nitrogen amount, biliprotein synthesis is directly linked and depends on the cell growth status: the more advanced the culture, the more the biliprotein content increases [51,65,66]. There are, however, some studies that report increased phycocyanin production induced by high illumination [35,67], but we feel they need further verification. In the present study, and especially in the case of *Phormidium,* beyond the middle of the exponential phase (seventh day), the low-intensity colored lights induced a profound increase of biliproteins in comparison with the respective amounts in low white light (40 μmol/m^2^/s), and the respective ones were tremendously diminished in high white light (160 μmol/m^2^/s), corroborating the similar findings of Alberte et al., 1984 [68], for *Synechococcus* concerning phycoerythrin. Our results for phycocyanin and phycoerythrin production yield in *Phormidium* at low illumination of 37 and 5.7 mg/g dw and 28 and 1.9 mg/g dw, respectively, for *Cyanothece*, are quite close to those for *Nostoc* sp. [51], where at 40 μmol/m^2^/s of white light and at high temperature of ~30 °C, total biliproteins accounted for 43 mg/g dw. In another study on the optimization of phycocyanin production in *Phormidium ceylanicum* [69] at ~27 °C and 130 μmol/m^2^/s of white light, the amount obtained was 0.32 mg/mL and enhanced to 0.73 mg/mL after optimization with the addition of certain chemicals. Furthermore, at a high temperature (28 °C) and at three light intensities of 80, 120 and 160 μmol/m^2^/s white light, in *Oscillatoria* sp. [62], the results indicated a rather uniform output between light intensities of phycoerythrin (~0.05 mg/mL), phycocyanin (~0.24 mg/mL) and allophycocyanin (~0.23 mg/mL) after 15 days of cultivation and with a biomass-specific growth rate of 0.228 doubl./day. In another study [70], *Spirulina platensis* in mixotrophic culture produced 2.38 g/L biomass and 0.261 mg/mL phycocyanin, while under continuous supplementation of CO_2_, it reached 7.27 g/L and 1.22 mg/mL biomass and phycocyanin, respectively [71]. Quite similar results were recorded for *Anabaena*, *Nostoc* and *Spirulina* species [72]. Our results for the maximum phycocyanin content of 0.32 mg/mL for both *Phormidium* and *Cyanothece* after 14 and 7 days, respectively, at ~19 °C and 40 μmol/m^2^/s and without the addition of CO_2_ are very much in accordance with them and very encouraging for the potential of further maximization using solely white light.

It is very important, mainly from the production perspective, to monitor the cellular content of pigments in order to decide when it is best to collect the biomass and extract it. The sooner the desired pigment content peaks, the better for the economics of the culture. Usually, the peak occurs somewhere in the exponential phase of growth between the 10th and 14th day (e.g., [62]), but it can be protracted for more than 30 days (e.g., [73]). From the data of various studies in the literature, no clear pattern can be drawn. The production of all kinds of pigments, among them chlorophyll, carotenoids or biliproteins, exhibits great variation on the time that they are maximized among species, the particular the pigment measured, conditions of culture and equipment used. In the present study, the culture period lasted 18 days for *Cyanothece* and 21 days for *Phormidium*, and we decided to end the cultures on these days as the concentration of most biliproteins had already peaked some days previously and in spite of the impressive fact that chlorophyll and carotenoids still were increasing. It is also noteworthy that among the biliproteins, allophycocyanin peaked earlier than phycocyanin and phycoerythrin in the culture of *Phormidium*, and although several studies refer to allophycocyanin (and also phycoerythrin) as the least in cellular content compared to phycocyanin [14,17,74], while in Patel et al., 2005 [75], phycocyanin and allophycocyanin are the main biliproteins in *Phormidium*, *Spirulina* and *Lyngbya*, in the present study, the allophycocyanin content exceeded that of phycocyanin in *Phormidium* and was close to half the concentration of phycocyanin in *Cyanothece* (all the cases refer to white light of 40 μmol/m^2^/s). This finding implies that diversification of photosynthetic pigments’ content among cyanobacterial species is a fact, at least between filamentous and single-celled (coccoid form). This is presumably one of the factors that contributes to the breakdown of nitrogen rich chlorophyll and biliproteins during the late phases of culture in high illumination, where only carotenoids, which are non-nitrogenous, are retained and, as a consequence, the coloration of the culture becomes yellowish [76].

Considering the influence of monochromatic light on the composition of phycobilisomes of different species of cyanobacteria that is manifested as complementary chromatic adaptation purposed to maximize the capture of available light [8,39], the results of the present study revealed that this phenomenon was observed in both *Phormidium* and *Cyanothece*, but with great differences among them, not only in the biliprotein amount, but in the biomass produced as well. In *Phormidium*, by far, the greatest amount of biomass was steadily induced by white light of 160 μmol/m^2^/s from the 14th day (1.45 g dw/L), peaking on the 21st day (2.15 g dw/L), while the relevant values of colored cultures were half of the above. The situation of the colored-light-induced fluctuation of biliprotein content along the culture period was completely opposite, where, after the seventh day, all colors used gave far greater amounts of biliproteins than those of low or high white light. In *Cyanothece*, this phenomenon was less pronounced than in *Phormidium* in the case of biomass produced by white light because colored light and especially green reached values similar to the white light values of ~1.68 g dw/L on the 18th day. However, considering the effect of colors on the biliprotein content, *Cyanothece* exhibited a profound supremacy of biliprotein production in every color used over white light from the ninth day. Contrary to some reports that consider blue light as less photosynthetically efficient for cyanobacterial growth compared to eukaryotic algae [31,77], in our study, blue light greatly enhanced phycoerythrin and allophycocyanin production (and by no means negligibly, phycocyanin) in *Phormidium*, while in *Cyanothece*, it induced the maximum phycocyanin production (similar with red and green), while phycoerythrin and allophycocyanin were by far maximized under green light. Other studies however indicate blue as the most suitable light for phycocyanin concentration [29,36,37,77], but not for promoting biomass, and this is partly in accordance with our findings for *Cyanothece*. Similar to the profound accumulation of phycoerythrin under green light in *Cyanothece*, this was recorded in *Fremyella diplosiphon* [78], while red light induced phycocyanin maximization. Red light induced enhanced phycocyanin production in both *Phormidium* and *Cyanothece*, but not to a lesser degree to blue light as in *Synechococcus* [28].

Reviewing the literature on the effect of light color on growth and biliprotein content among cyanobacterial cultures, there are so many perplexing data that no solid conclusion can be reached, nor is it possible to manifest a guideline for maximizing the production of either biomass or (mainly) biliproteins. As an example of this stands the following. Green light was found most suitable for phycocyanin and allophycocyanin in *Nostoc* [51] and for phycoerythrin in *Fremyella diplosiphon, Calothrix, Gloeotrichia* and *Pseudoanabaena* [60,79,80], but not suitable for the growth of *Synechococcus* [58]. Green light along with blue were found also to maximize total photosynthetic pigments (chlorophyll included) in marine *Synechococcus* sp. [81]. Blue light enhances the phycocyanin to chlorophyll ratio in *Synechocystis* sp. [77], biomass and phycocyanin in *Arthrospira platensis* along with red light [31], phycocyanin in *Synechococcus* sp. [8], biomass in *Pseudoanabaena* [60], total biliproteins in *Anabaena ambigua* [46], *Westellopsis iyengarii* [82], *Spirulina fussiformis* [59] and *Nostoc sphaeroides* [83] and phycoerythrin in *Calothrix elenkinii* [80]. The minimum synthesis of phycocyanin and allophycocyanin was however reported in *Nostoc* sp. [51]. Red light enhances phycocyanin synthesis in *Calothrix elenkinii* [80], *Fremyella diplosiphon*, *Calothrix* sp. and *Spirulina platensis* [28,55,79] and *Pseudoanabaena* sp. [60], while inhibiting phycoerythrin synthesis in *Gloeotrichia* sp. and *Nostoc* sp. [79], maximum growth in *Synechococcus* sp. [58] and total biliproteins in *Anacystis nidulans* [84], *Synechococcus* sp. [63], *Calothrix* sp. [85] and *Nostoc* spp. [86,87].

Concerning the universally present chlorophyll-a and carotenoids in all photosynthetic algae, our study found that green light greatly enhanced the chlorophyll content in *Phormidium* after 14 days of culture onward, and the same occurred in *Cyanothece*, but to a lesser degree. In *Cyanothece*, white light rivaled green light in enhancing chlorophyll content, and this occurred to a lesser degree in *Phormidium*. Chlorophyll content was more in white light of low intensity as compared to high intensity in the case of *Phormidium*, and this is in accordance with the similar trend found in *Anabaena ambigua* [46]. White light of high intensity (160 μmol/m^2^/s) by far exceeded all colors in enhancing total carotenoids and b-carotene in *Phormidium*, and the same, but to a lesser degree occurred in *Cyanothece*. Very limited information can be found in the literature about the effect of colored light on the cyanobacterial chlorophyll and carotenoids content as the majority of studies are focused on biliproteins. Even so, an indirect clue about the variation of chlorophyll content among cyanobacteria is the ratio of biliproteins to chlorophyll, which depends on the color of light in which they are growing [76]. In this respect, by far the biggest values among all colors recorded, blue and red colors in *Phormidium* (~600:1) reflect the compensatory adjustment of cells at these wavelengths, where chlorophylls absorb the maximum, but biliproteins cannot. Green light affected maximum chlorophyll content in *Phormidium* (9.3 μg/mL or 13.75 mg/g dw) and *Cyanothece* (11.55 mg/g dw) from the 14th day onwards, quite similar to what has been reported for *Westelliopsis prolifica, Nostoc muscorum, Aulosira fertilissima, Westelliopsis fertilissima, Tolypothrix tenuis* and *Anabaena variabilis* [88], but differing from them in the respect that they did not mention either the kind of light or the conditions of culture used, and in our cultures, there was not a steep drop of values after the 14th day, as they reported. Similar values of chlorophyll to ours are also reported for *Limnothrix redekei* and *Planktothrix agardhii* (9.95 and 6.07 mg/g dw, respectively) using white light [89], while much reduced values of 1.6 and 2.0 mg/g dw of chlorophyll-a and chlorophyll-b, respectively, in green light were given for *Arthrospira platensis* by [90]; however, their value of chlorophyll-b should be rejected, as, with the exception of Prochlorophytes, cyanobacteria do not contain this pigment. Cultures of *Nostoc calcicola* [91] at 25 °C and under low-intensity white light (20–60 μmol/m^2^/s) gave 0.8–1.1 g dw/L, 3–4 mg/g dw chlorophyll-a and 6–8 mg/g dw total carotenoids, but the narrow range of light intensity they used and the absence of implementation of colored light does not allow for a sound conclusion about the influence on pigment content by either the intensity of light or of its color. Total carotenoids in the present study in *Phormidium* was much enhanced by high-intensity white light (4.2 μg/mL on the 21st day), almost double that for low intensity (2.36 μg/mL) and close to 3 μg/mL for green and red light. Lower values as compared to *Phormidium* were recorded in *Cyanothece* in high white light (2.97 μg/mL on the 18th day) and 2.7 μg/mL in green light. Transforming these values into mg/g dw, our results of total carotenoids with values of 2.3–3.1 mg/g dw for colored light and ~2.0 mg/g dw for white light in *Phormidium* and about 20% lower in *Cyanothece* are much higher than the respective ones for the five cyanobacteria recorded by [88]. The ratio of total carotenoids to b-carotene in *Phormidium* remained quite uniform in all light treatments between 3:1 and 4.4:1 with maximum concentrations of b-carotene in high white light (0.95 mg/L), followed by green light (0.78 mg/L). Although we did not find relative studies in the literature concerning cyanobacterial b-carotene content, we assumed that our results are very promising in terms of production as compared to the green algae *Dunaliella salina,* notoriously known for the accumulation of b-carotene, which, under very intense light (~1500 μmol/m^2^/s), can yield ~1–3 mg/L [92]. More research is needed to deepen the understanding of the influence of light on the pigment content in cyanobacteria, especially in combination with other factors that may substantially influence their content. Overall, it was clear in the present experiment that algal mass is maximized by white light and phycobiliproteins by colored light. A sound scenario to obtain the most benefit from culturing these two cyanobacteria is to culture them in two steps: first, under white light of about 160 μmol/m^2^/sμmol/m^2^/s and with a supply of extra CO_2_, which can greatly enhance growth [93] in order to maximize biomass, and next, subjection to colored light in order to maximize phycobiliproteins.

## 5. Conclusions

The local strains of *Phormidium* sp. and *Cyanothece* sp. are good candidate cyanobacterial species for mass cultivation and pigment production, as they produce considerably amounts of dry biomass (2.1 g dw/L for *Phormidium*, 1.47 g dw/L for *Cyanothece*) even at the mediocre temperature of 18–19.5 °C. These values rank among the highest for exploitable cyanobacteria. White light and especially that of 160 μmol/m^2^/s maximize biomass yield, while colored light promotes phycobiliprotein content in both species, and although there is not a definite pattern to differentiate among blue, green and red regarding their effect on pigment content, it is green light that seems superior to other colors in elevating phycobiliproteins, chlorophyll and carotenoids content in both cyanobacteria. Overall, a production of >30 mg/g dw phycocyanin and >12 mg/g dw phycoerythrin can easily be produced by culturing both species using the appropriate colored light, and these yields can probably be greatly increased using a temperature higher than 19 °C, the addition of pure CO_2_, the addition of special nutrients and photobioreactor technology.

## Figures and Tables

**Table 1:** Descriptive statistics of the specific growth rate (SGR in doubl./day) and generation time (Tg in days) of different variables across light treatments in both *Phormidium* (suffix: Ph-) and *Cyanothece* (suffix: Cy-) *. W = white light, L = 40 μmol/m^2^/s, XL = 160 μmol/m^2^/s, BLUE, GREEN, RED = colors of light used. For *Phormidium*, the 7th–14th day interval was used and for *Cyanothece* 9th–15th.

Mean Values ± SE	Ph-W-XL	Ph-W-L	Ph-BLUE	Ph-GREEN	Ph-RED	Cy-W-XL	Cy-W-L	Cy-BLUE	Cy-GREEN	Cy-RED
SGR (doubl./d)	0.085 ^a^ ±0.0003	0.062 ^b^ ±0.0004	0.041 ^c,e^ ±0.0005	0.102 ^d^ ±0.0004	0.044 ^e,c^ ±0.0009	0.083 ^f^ ±0.001	0.038 ^g^ ±0.0002	0.037 ^h^ ±0.0007	0.023 ^i^ ±0.0005	0.019 ^j^ ±0.0016
Tg (days)	8.16	11.12	17.06	6.78	15.7	8.37	18.35	18.6	30.64	35.92

* Values are means ± S.E. (standard error) of 3 measurements. The different superscripts (a, b, c, d, e, f, g, h, i, j) indicate a statistically significant difference at the 0.05 level of confidence (statistical processing with ANOVA and then pairwise comparison with Tukey’s test). Where there is a second superscript, it means statistically equal to the value of the condition of the corresponding letter.

**Table 2:** Descriptive statistics of maximal values of different variables across light treatments in both *Phormidium* (suffix: Ph-) and *Cyanothece* (suffix: Cy-) *. W = white light, L = 40 μmol/m^2^/s, XL = 160 μmol/m^2^/s, BLUE, GREEN, RED = colors of light used. Chl.-a = chlorophyll-a, Tcar = total carotenoids, PC = phycocyanin, PE = phycoerythrin, APC = allophycocyanin, PBP = phycobiliproteins.

Max. Values ± SE	Ph-BLUE	Ph-GREEN	Ph-RED	Ph-W-XL	Ph-W-L	Cy-BLUE	Cy-GREEN	Cy-RED	Cy-W-XL	Cy-W-L
Chl.-a (μg/mL)	4.08 ^c^ ±0.023	9.3 ^d^ ±0.07	6.85 ^e^ ±0.034	4.65 ^a,h^ ±0.08	5.93 ^b^ ±0.045	4.7 ^h,a^ ±0.078	9.31 ^i,d,g^ ±0.13	3.67 ^j^ ±0.05	7.47 ^f^ ±0.11	8.94 ^g,d,i^ ±0.6
Tcar (μg/mL)	2.14 ^c^ ±0.03	2.8 ^d^ ±0.021	2.66 ^e^ ±0.006	4.9 ^a^ ±0.05	2.36 ^b^ ±0.006	1.54 ^h, j^ ±0.0261	2.71 ^i^ ±0.036	1.55 ^j, h^ ±0.0261	2.97 ^f^ ±0.025	0.96 ^g^ ±0.0115
PC (mg/mL)	0.31 ^c, b^ ±0.009	0.447 ^d^ ±0.003	0.315 ^e,b,c,j^ ±0.0065	0.256 ^a^ ±0.006	0.317 ^b,c^ ±0.0004	0.305 ^h,b,c,g^ ±0.0025	0.296 ^i^ ±0.0147	0.327 ^j,b,c,e,g^ ±0.0148	0.195 ^f^ ±0.0134	0.321 ^g,b,c^ ±0.025
PE (mg/mL)	0.175 ^c^ ±0.002	0.147 ^d^ ±0.004	0.138 ^e,i^ ±0.003	0.022 ^a^ ±0.001	0.1 ^b^ ±0.0001	0.0017 ^h^ ±0.00007	0.136 ^i,e^ ±0.0014	0.015 ^j^ ±0.0002	0.016 ^f^ ±0.00031	0.02 ^g^ ±0.00038
APC (mg/mL)	0.824 ^c^ ±0.008	0.78 ^d^ ±0.003	0.751 ^e^ ±0.005	0.134 ^a,h^ ±0.003	0.51 ^b,i^ ±0.056	0.131 ^h,a^ ±0.0005	0.371 ^i,b^ ±0.0056	0.121 ^j^ ±0.0008	0.092 ^f^ ±0.0015	0.165 ^g^ ±0.0035
Total PBP (mg/mL)	1.084	1.145	0.940	0.403	0.854	0.437	0.768	0.452	0.303	0.506
PCyield (mg/g dw)	18.52 ^c,a,f,h^ ±0.71	31.76 ^d^ ±0.48	22.54 ^e^ ±0.47	19.41 ^a,c,f,h^ ±0.44	20.68 ^b^ ±0.37	19.26 ^h,a,c,f^ ±0.89	27.43 ^i^ ±0.98	34.6 ^j^ ±0.88	18.34 ^f,a,c^ ±0.98	29.16 ^g^ ±1.95
PEyield (mg/g dw)	10.62 ^c^ ±0.11	10.44 ^d^ ±0.005	10.87 ^e^ ±0.057	1.09 ^a^ ±0.0006	6.091 ^b^ ±0.005	0.122 ^h^ ±0.0015	12.07 ^i^ ±1.031	0.151 ^j^ ±0.0137	1.16 ^f^ ±0.075	1.83 ^g^ ±0.078
Chl.: Tcar	4.18	5.44	5.04	2.2	3.99	3.04	3.9	3.01	4.1	15.24
PBP: Chl.-a	616	428	620	106	173	109	187	109	47	62
PBP:Tcar	1735	2096	3028	233	690	331	632	371	190	938
PC: PE	40.3	39.3	8.4	20	18.1	304	15	67	150	174
PC: APC	1.4	1.6	1.7	2.0	2.0	2.77	2.71	3.51	2.12	2.12

* Values are means ± S.E. (standard error) of 3 measurements. The different superscripts (a, b, c, d, e, f, g, h, i, j) indicate a statistically significant difference at the 0.05 level of confidence (statistical processing with ANOVA and then pairwise comparison with Tukey’s test). Where there is a second superscript, it means statistically equal to the value of the condition of the corresponding letter.

## Supplementary Materials

The following supporting information can be downloaded at: https://www.mdpi.com/article/10.3390/life12060837/s1, Supplementary Material S1: calibration curves and growth curves of *Phormidium* sp. and *Cyanothece* sp. cultures.
